# Effect of bevacizumab combined with boron neutron capture therapy on local tumor response and lung metastasis

**DOI:** 10.3892/etm.2014.1704

**Published:** 2014-05-08

**Authors:** SHIN-ICHIRO MASUNAGA, YOSHINORI SAKURAI, KEIZO TANO, HIROKI TANAKA, MINORU SUZUKI, NATSUKO KONDO, MASARU NARABAYASHI, TSUBASA WATANABE, YOSUKE NAKAGAWA, AKIRA MARUHASHI, KOJI ONO

**Affiliations:** Department of Radiation Life and Medical Science, Research Reactor Institute, Kyoto University, Osaka 590-0494, Japan

**Keywords:** bevacizumab, boron neutron capture therapy, ^10^B-carrier, quiescent cell, nicotinamide, mild temperature hyperthermia

## Abstract

The aim of the present study was to evaluate the effect of bevacizumab on local tumor response and lung metastatic potential during boron neutron capture therapy (BNCT) and in particular, the response of intratumor quiescent (Q) cells. B16-BL6 melanoma tumor-bearing C57BL/6 mice were continuously administered bromodeoxyuridine (BrdU) to label all proliferating (P) tumor cells. The tumors were irradiated with thermal neutron beams following the administration of a ^10^B-carrier [L-*para*-boronophenylalanine-^10^B (BPA) or sodium mercaptoundecahydrododecaborate-^10^B (BSH)], with or without the administration of bevacizumab. This was further combined with an acute hypoxia-releasing agent (nicotinamide) or mild temperature hyperthermia (MTH, 40°C for 60 min). Immediately following the irradiation, cells from certain tumors were isolated and incubated with a cytokinesis blocker. The responses of the Q cells and the total (P+Q) cell populations were assessed based on the frequency of micronuclei using immunofluorescence staining for BrdU. In other tumor-bearing mice, 17 days following irradiation, lung metastases were enumerated. Three days following bevacizumab administration, the sensitivity of the total tumor cell population following BPA-BNCT had increased more than that following BSH-BNCT. The combination with MTH, but not with nicotinamide, further enhanced total tumor cell population sensitivity. Regardless of the presence of a ^10^B-carrier, MTH enhanced the sensitivity of the Q cell population. Regardless of irradiation, the administration of bevacizumab, as well as nicotinamide treatment, demonstrated certain potential in reducing the number of lung metastases especially in BPA-BNCT compared with BSH-BNCT. Thus, the current study revealed that BNCT combined with bevacizumab has the potential to sensitize total tumor cells and cause a reduction in the number of lung metastases to a similar level as nicotinamide.

## Introduction

The neutron capture reaction in boron [^10^B(n,α)^7^Li] is, in principle, very effective in destroying tumors, provided that a sufficient amount of ^10^B is accumulated in the target tumor and a sufficient number of very-low-energy thermal neutrons are delivered. The two particles generated in this reaction have a high linear energy transfer (LET) and have a range of ~1–2 tumor cells in diameter. It is theoretically possible to destroy tumor cells without affecting adjacent healthy cells if ^10^B atoms are selectively accumulated in the interstitial space of tumor tissue and/or intracellular space of tumor cells. Thus, successful boron neutron capture therapy (BNCT) requires the selective delivery of large amounts of ^10^B to tumor cells (1).

The two most common ^10^B-carriers used in clinical BNCT trials, designed for the treatment of malignant gliomas, melanomas, inoperable head and neck tumors, and oral cancer, are sodium mercaptoundecahydrododecaborate-^10^B (BSH, Na_2_^10^B_12_H_11_SH) and L-*para*-boronophenylalanine-^10^B (BPA, C_9_H_12_^10^BNO_4_) (1). The delivery of ^10^B via BSH relies on passive diffusion from the blood to the brain tumor through a disrupted blood-brain barrier (2). Thus, the use of BSH results in a high concentration of boron in the blood and subsequent vascular damage during BNCT (3). BPA is designed to be taken up mainly by active transport across the cancer cell membrane (4). However, the transport mechanism is operative even in normal cells leading to the accumulation of BPA in normal brain tissues, although at a lower rate. Therefore, it has been suggested that tumor response may be improved by combining BSH and BPA (5).

Antiangiogenic therapy has been hypothesized to prevent vascular tumor growth and proliferation, thus depriving the tumor of the oxygen and nutrients necessary for survival (6). However, a subsequent study suggested that antiangiogenic therapy may also ‘normalize’ the tumor vasculature for a short period of time, thereby providing a window of opportunity for improved drug delivery and enhanced sensitivity to radiation (6,7). Tumor hypoxia results from either limited oxygen diffusion (chronic hypoxia) or limited perfusion (acute hypoxia) (8). Previous studies have reported that acute and cyclic, but not chronic, hypoxia significantly increased the number of spontaneous lung metastases and that this effect was due to the influence of acute hypoxia treatment on the primary tumor (9,10).

In the present study, the efficiency of administering the vascular endothelial growth factor (VEGF) inhibitor, bevacizumab, in combination with BNCT, and further combined with the acute hypoxia-releasing agent, nicotinamide, or mild temperature hyperthermia (MTH) was evaluated. MTH has already demonstrated the potential to release tumor cells from diffusion-limited chronic hypoxia (11,12) in terms of local tumor response and lung metastasis. In addition, with regards to the local tumor response, the effect not only on the total tumor cell population [proliferating (P) + quiescent (Q)], but also on the Q cell population alone, was evaluated using an original method for selectively detecting the response of Q cells in solid tumors (13).

## Materials and methods

### Mice and tumors

B16-BL6 murine melanoma cells (Institute of Development, Aging and Cancer, Tohoku University, Sendai, Japan) derived from C57BL/6 mice were maintained *in vitro* in RPMI-1640 medium supplemented with 10% fetal bovine serum. Tumor cells (1.25×10^5^) were inoculated subcutaneously into the left hind leg of 8-week-old syngeneic female C57BL/6 mice (Japan Animal Co., Ltd., Osaka, Japan). Eighteen days later, the tumors, ~7 mm in diameter, were employed for treatment. The body weight of the tumor-bearing mice was 20.1±2.3 g (mean ± standard error). The mice were handled according to the Recommendations for Handling of Laboratory Animals for Biomedical Research, compiled by the Committee on Safety Handling Regulations for Laboratory Animal Experiments at Tohoku University. The p53 of the B16-BL6 tumor cells was the wild-type (14).

### Labeling with bromodeoxyuridine (BrdU)

Twelve days following inoculation, mini-osmotic pumps (Durect Corporation, Cupertino, CA, USA) containing BrdU dissolved in physiological saline (250 mg/ml) were implanted subcutaneously into the backs of the animals for six days in order to label all P cells. The percentage of labeled cells following the continuous treatment with BrdU reached a plateau at this stage. Therefore, tumor cells not incorporating BrdU following continuous labeling were regarded as Q cells.

### Treatment

Fifteen days following the tumor cell inoculation, bevacizumab (a humanized monoclonal antibody against VEGF; Hoffmann-La Roche AG, Basel, Switzerland) dissolved in physiological saline was intravenously administered through a tail vein, at a dose of 10 mg/kg in a single injection. Bevacizumab has previously been demonstrated to induce a period of vascular normalization in B16-F10 murine melanoma tumors originating from B16-F1 murine melanoma cells (15). Thus, in B16-BL6 tumors also originating from B16-F1 murine melanoma cells, it was hypothesized that bevacizumab would exhibit the same effect as in B16-F10 tumors. After three days (18 days following inoculation, on day 18), the percentages of labeled cells following the continuous administration of BrdU for six days were 60.1±6.8 and 54.3±6.1% for those treated with bevacizumab and those not, respectively.

BSH and BPA were purchased from KatChem Ltd. (Prague, Czech Republic), and prepared by dissolving in physiological saline for BSH (125 mg/kg) and as a complex with 3% fructose for BPA (250 mg/kg). They were injected intraperitoneally in a volume of 0.02 ml/g of mouse body weight. In accordance with previous studies (16), no overt toxicity was observed at a dose of <500 mg/kg for BSH and <1,500 mg/kg for BPA. Based on the certificate of analysis and material safety data sheet, provided by the manufacturer, borocaptate dimer (BSSB; ^10^B_24_H_22_S_2_^4−^) was not present as a contaminant. The intratumor ^10^B concentration during neutron irradiation is a crucial determinant of the cell destruction effect of BNCT. In order to obtain similar intratumor ^10^B concentrations during exposure to the neutron beam, irradiation was initiated at selected time points following the intraperitoneal injection of the ^10^B-carriers at a selected dose of ^10^B. Based on a preliminary study of the biodistribution of ^10^B, irradiation was initiated from 60 min following intraperitoneal injection of 125 and 250 mg/kg (71.0 and 12.0 mg ^10^B/kg) of BSH and BPA, respectively. ^10^B concentrations were determined using a thermal neutron guide tube installed at the Kyoto University Research Reactor (Osaka, Japan) (17).

Certain tumor-bearing mice also received an intraperitoneal administration of nicotinamide (1,000 mg/kg) dissolved in physiological saline 1 h prior to neutron irradiation. Others were subjected to local MTH at 40°C for 1 h by immersing the implanted tumor in a water bath prior to irradiation (18). Temperatures in the center of the tumors equilibrated within 3–4 min following immersion in the water bath and remained at 0.2–0.3°C below the temperature of the bath. The water temperature in the bath was maintained at 0.3°C above the desired tumor temperature (18).

For irradiation of the tumors implanted into the left hind legs of the mice, a device composed of acrylic resin and capable of holding 12 mice was used. The tumor-bearing mice were irradiated with a reactor neutron beam at a power of 1 MW at Kyoto University Research Reactor after being fixed in position with adhesive tape. A lithium fluoride (LiF) thermoplastic shield was employed to avoid irradiating body parts other than the implanted solid tumors. Neutron irradiation was performed using a reactor neutron beam with a cadmium ratio of 9.4. The neutron fluence was measured from the radioactivation of gold foil at both the front and back of the tumors. Since the tumors were small and located just beneath the skin surface, the neutron fluence was assumed to decrease linearly from the front to the back of the tumors. Thus, the average neutron fluence, determined from the values measured at the front and back of the tumor, was used. Contaminating γ-ray, including secondary γ-ray, doses were measured with a thermoluminescence dosimeter (TLD) powder placed at the back of the tumors. The TLD used was beryllium oxide (BeO) enclosed in a quartz glass capsule (Panasonic Corporation, Osaka, Japan). The BeO itself was not sensitive to thermal neutrons. The thermal neutron fluence, of 8×10^12^/cm^2^, was equal to ~1 cGy γ-ray dose. TLD was normally used together with the gold activation foil for the neutron-sensitivity correction in the current study. The details were described in the study by Sakurai and Kobayashi (19). For the estimation of neutron energy spectra, eight types of activation foil and 14 types of nuclear reaction were used (19). The absorbed dose was calculated using the flux-to-dose conversion factor (20). The tumors contained H (10.7% in terms of weight), C (12.1%), N (2%), O (71.4%), and others (3.8%) (21). The average neutron flux and kerma rate of the employed beam were 1.0×10^9^ n/cm/sec and 48.0 cGy/h for the thermal neutron range (<0.6 eV), 1.6×10^8^ n/cm/sec and 4.6 cGy/h for the epithermal neutron range (0.6–10 keV), and 9.4×10^6^ n/cm/sec and 32.0 cGy/h for the fast neutron range (>10 keV), respectively. The kerma rate for boron dose per φ n/cm/s of thermal neutron flux for 1 μg/g of ^10^B was 2.67×10–^8^φ cGy/h. The contaminating γ-ray dose rate was 66.0 cGy/h.

Each irradiation group also included mice that were not pre-treated with BrdU.

### Immunofluorescence staining of BrdU-labeled cells and micronucleus (MN) assays

Immediately following irradiation, selected tumors were removed from the mice given BrdU. They were homogenized and trypsinized using 0.05% trypsin and 0.02% ethylenediaminetetraacetic acid (EDTA) in phosphate-buffered saline (PBS) at 37°C for 15 min. Tumor cell suspensions were incubated for 72 h in tissue culture dishes containing complete medium and 1.0 μg/ml of cytochalasin B in order to inhibit cytokinesis while allowing nuclear division. The cultures were trypsinized and cell suspensions were fixed and resuspended with cold Carnoy’s fixative (ethanol:acetic acid, 3:1 by volume). Each suspension was placed on a glass microscope slide, dried at room temperature and treated with 2 M hydrochloric acid for 60 min at room temperature in order to dissociate the histones and partially denature the DNA. The slides were immersed in borax-borate buffer (pH 8.5) to neutralize the acid. BrdU-labeled tumor cells were detected by indirect immunofluorescence staining using a monoclonal anti-BrdU antibody (BD Biosciences, San Jose, CA, USA) and a fluorescein isothiocyanate (FITC)-conjugated antimouse IgG antibody (Sigma, St. Louis, MO, USA). To distinguish the tumor cells stained with green-emitting FITC and observe them separately, cells on the slides were treated with red-emitting propidium iodide (PI; 2 μg/ml in PBS) as a background stain and monitored under a fluorescence microscope (Olympus Bioimaging, Tokyo, Japan).

When cell division is disrupted, or the chromosomes are broken or damaged by chemicals or radiation, the distribution of genetic material between the two daughter nuclei during cell division is affected and pieces or entire chromosomes fail to be included in either of the two daughter nuclei. The genetic material that is not incorporated into a new nucleus forms a ‘MN’. Thus, the frequency of MN formation accurately reflects the genotoxicity of a chemical compound and radiation. The MN frequency in cells not labeled with BrdU was examined by counting the micronuclei in the binuclear cells that showed only red fluorescence. The MN frequency was defined as the ratio of the number of micronuclei in the binuclear cells to the total number of binuclear cells observed (13).

The ratios obtained from the tumors not pretreated with BrdU indicated the MN frequencies at all phases in the total tumor cell population. More than 300 binuclear cells were counted to determine the MN frequency.

### Clonogenic cell survival assay

Immediately following irradiation, a clonogenic cell survival assay was performed on the implanted tumors in mice that were not given BrdU using an *in vivo-in vitro* assay method. The BrdU-unlabeled tumors were removed, weighed, homogenized and disaggregated by stirring for 20 min at 37°C in PBS containing 0.05 % trypsin and 0.02% EDTA. The cell yield was 1.2±0.4×10^7^/g tumor weight. Appropriate numbers of viable tumor cells from the single cell suspension were plated on 60- or 100-mm tissue culture dishes and 12 days later, colonies were fixed with ethanol, stained with Giemsa and counted. For the tumors that received no irradiation, plating efficiencies for the total tumor cell populations and the MN frequencies for the total and Q cell populations are displayed in Table I. The plating efficiency indicates the percentage of cells seeded that grew into colonies when the tumors received no irradiation. The fraction of cells surviving a given dose was determined by counting the number of macroscopic colonies as a fraction of the total number of cells seeded, followed by allowance, that is, by dividing by the plating efficiency.

As stated above, the MN frequencies for Q cells were obtained from BrdU-unlabeled cells in tumors following continuous BrdU labeling *in vivo*. The MN frequencies and surviving fractions (SFs) for the total tumor cell populations were obtained from cells in tumors not pretreated with BrdU. Thus, the present study was not able to detect any interaction between BrdU and irradiation in the data for the MN frequencies and SFs.

### Metastasis assessment

Seventeen days following irradiation (35 days following the inoculation of B16-BL6 melanoma cells), the tumor-bearing mice were sacrificed by cervical dislocation. Their lungs were removed, briefly washed with distilled water, cleaned of extraneous tissue, fixed overnight in Bouin’s solution (Sigma), and stored in buffered formalin 10% (Sigma) until metastases were counted. Macroscopically visible metastases were counted using a dissection microscope (22). Eighteen days following inoculation and immediately prior to exposure to the neutron beam, macroscopic lung metastases were also counted as background data; the number was 7.5±2.2.

### Data analysis and statistics

Three mice with a tumor in the left hind leg were used to assess each set of conditions and each experiment was repeated three times. Thus, in total, nine mice were used for each set of conditions. To examine the differences between pairs of values, the Student’s t-test was used when variances of the two groups were assumed to be equal; otherwise the Welch’s t-test was used. P-values were obtained from two-sided tests. P<0.05 was considered to indicate a statistically significant difference. The data on cell survival and MN frequencies were fitted to the linear-quadratic dose relationship (23).

## Results

### Toxicity of ^10^B-carriers and bevacizumab

Table I shows the plating efficiencies for the total tumor cell population and the MN frequencies without irradiation for the total and Q cell populations. The Q cell population revealed significantly higher MN frequencies than the total cell population under each set of conditions (P<0.05). The combination with bevacizumab increased the sensitivity of the total cells more than that of the Q cells, especially when BPA was employed compared with BSH or no ^10^B-carrier. The difference, however, was not significant.

### ^10^B concentrations and ^10^B dose rate during irradiation

Table II shows the ^10^B concentrations and boron dose rates in irradiated tumors for each set of conditions. The values are averages obtained using the ^10^B concentrations at the start and end points of the irradiation time. The combination with bevacizumab increased the concentration, especially when BPA was employed compared with BSH. When BPA was used as a ^10^B-carrier, MTH increased the concentration more than nicotinamide. By contrast, with BSH as the ^10^B-carrier, nicotinamide increased the concentration more than MTH. However, again, these differences were not significant.

### Initial tumor response

**Figure 1:**
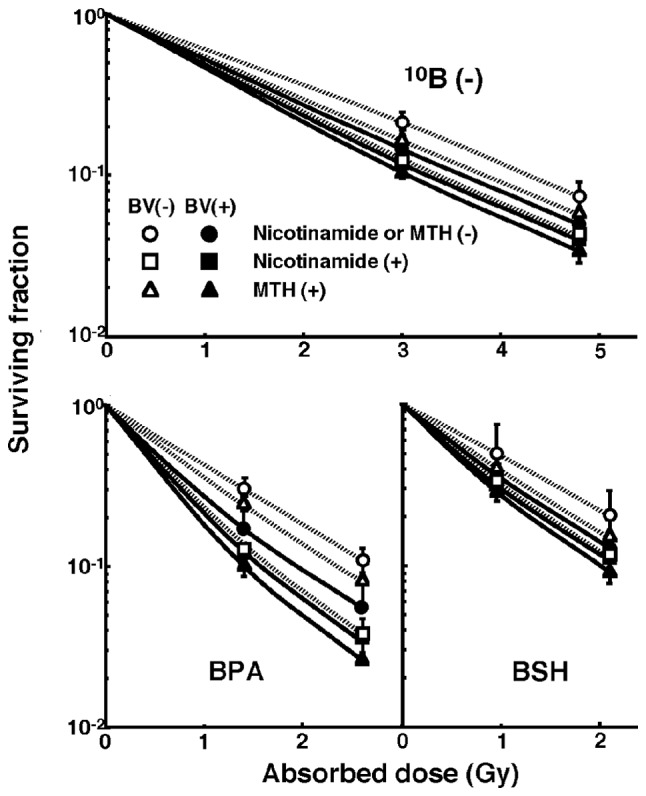
Cell survival curves for the total cell population from B16-BL6 tumors irradiated with reactor neutron beams following the administration of a ^10^B-carrier in combination with nicotinamide treatment, mild temperature hyperthermia (MTH), or bevacizumab treatment on day 18 following tumor cell inoculation. Open and solid symbols represent irradiation without and with bevacizumab, respectively. Circle, square, and triangle symbols represent irradiation without nicotinamide or MTH, with nicotinamide, and with MTH, respectively. Bars represent standard errors (n=9). ^10^B (−), no ^10^B-carrier; BPA, L-*para*-boronophenylalanine-^10^B; BSH, sodium mercaptoundecahydrododecaborate-^10^B; MTH, mild temperature hyperthermia; BV, bevacizumab.

**Figure 2:**
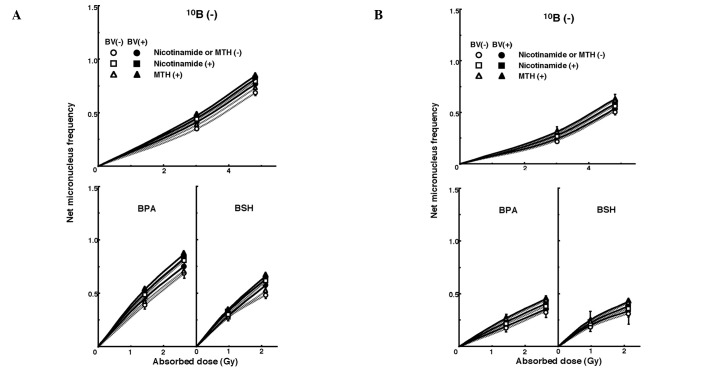
Dose response curves of the net micronucleus frequency for (A) total and (B) quiescent cell populations from B16-BL6 tumors irradiated with reactor neutron beams following the administration of a ^10^B-carrier in combination with nicotinamide treatment, mild temperature hyperthermia (MTH) or bevacizumab treatment on day 18 following tumor cell inoculation. Open and solid symbols represent irradiation without and with bevacizumab, respectively. Circle, square, and triangle symbols represent irradiation without nicotinamide or MTH, with nicotinamide, and with MTH, respectively. Bars represent standard errors (n=9). ^10^B (−), no ^10^B-carrier; BPA, L-*para*-boronophenylalanine-^10^B; BSH, sodium mercaptoundecahydrododecaborate-^10^B; MTH, mild temperature hyperthermia; BV, bevacizumab.

Fig. 1 shows cell survival curves for the total cell population as a function of the absorbed dose of neutron beam irradiation with or without a ^10^B-carrier, in combination with nicotinamide or MTH, and in the presence or absence of bevacizumab. Fig. 2 shows net MN frequencies as a function of irradiated absorbed dose with or without a ^10^B-carrier, in combination with nicotinamide or MTH, and in the presence or absence of bevacizumab in the total and Q tumor cell populations. The net MN frequency was the MN frequency in tumors that received irradiation minus the MN frequency in tumors that did not. Overall, the net MN frequencies were significantly smaller in Q cells than in the total cell population (P<0.05).

Lung metastases from local tumors. To estimate the radio-enhancing effect of the ^10^B-carriers, irradiation with BPA and BSH in both the total and Q cell populations was compared with neutron beam irradiation only, using the data obtained without nicotinamide or MTH shown in Figs. 1 and 2 (Table III). Both BPA and BSH enhanced the sensitivity of the total cell population significantly more than the Q cell population (P<0.05). Furthermore, BPA demonstrated a tendency to affect the sensitivity of the total cell population more than BSH. By contrast, the sensitivity of the Q cells was enhanced more by BSH than BPA. When bevacizumab was combined, the sensitivity of the total cells was enhanced more than that of the Q cells, especially when BPA was used compared with BSH. However, these differences were not statistically significant.

The data in Figs. 1 and 2 were used to estimate the radio-enhancing effect of combined treatment with bevacizumab in both the total and Q cell populations (Table IV). With or without a ^10^B-carrier, the sensitivity of the total cell population was enhanced when bevacizumab was administered, especially when BPA and/or MTH were combined. When nicotinamide and MTH were added, sensitivity was more suppressed and enhanced, respectively, in the total cells than in the Q cells. However, again, the differences were not significant.

The data in Figs. 1 and 2 were also used to estimate the radio-enhancing effect of combined treatment with nicotinamide or MTH in the total and Q cell populations (Table V). With BSH and without a ^10^B-carrier, the sensitivity of the total cell population was more enhanced with nicotinamide than with MTH. With BPA, the sensitivity of the total cell population was more enhanced with MTH than with nicotinamide. By contrast, the sensitivity of the Q cell population was more enhanced with MTH than with nicotinamide. Notably, with BPA or BSH as the ^10^B-carrier, MTH enhanced the sensitivity of the Q cell populations significantly more than the total cell populations (P<0.05). When bevacizumab was used, the enhancing effect of nicotinamide was suppressed, especially in the total cell population, although not significantly. However, the combination with bevacizumab revealed no significant effect on the enhancing effect of MTH.

To examine the difference in radio-sensitivity between the total and Q cell populations, dose-modifying factors were calculated using the data in Figs. 1 and 2 (Table VI). Overall, the values obtained were significantly >1.0 (P<0.05). Regardless of the ^10^B-carrier used, the difference in radio-sensitivity significantly increased (P<0.05), although the difference was smaller with BSH than with BPA. The difference in radio-sensitivity, especially without bevacizumab, was increased with nicotinamide and reduced with MTH. However, the difference following irradiation without nicotinamide or MTH or, in particular, with MTH, was increased with bevacizumab. By contrast, the difference following irradiation and nicotinamide administration was not significantly altered with bevacizumab.

### Lung metastases from local tumors

**Figure 3:**
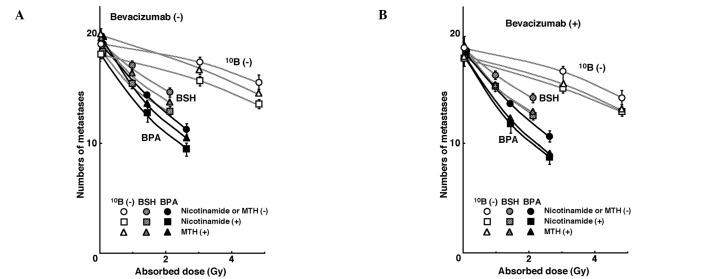
Counted numbers of macroscopic metastases in the lung on day 35 following tumor cell inoculation as a function of the dose of neutron beam irradiation (A) without and (B) with bevacizumab treatment following the administration of a ^10^B-carrier in combination with nicotinamide treatment or mild temperature hyperthermia (MTH) on day 18 following tumor cell inoculation. Circle, square, and triangle symbols represent irradiation without nicotinamide or MTH, with nicotinamide, and with MTH, respectively. Bars represent standard errors (n=9). ^10^B (−), no ^10^B-carrier; BPA, L-*para*-boronophenylalanine-^10^B; BSH, sodium mercaptoundecahydrododecaborate-^10^B; MTH, mild temperature hyperthermia.

Fig. 3 shows the numbers of lung metastases on day 35 following inoculation as a function of the absorbed dose of neutron beam irradiation with or without a ^10^B-carrier, in combination with nicotinamide or MTH, and in the presence or absence of bevacizumab treatment. Without bevacizumab or irradiation, irrespective of a ^10^B-carrier, nicotinamide and MTH decreased and increased the numbers of macroscopic metastases, respectively. With bevacizumab, but under no irradiation, both nicotinamide and MTH decreased the number of metastases. With neutron beam irradiation, as the absorbed dose increased, the number of metastases decreased. Furthermore, the number of metastases decreased markedly with a ^10^B-carrier, especially BPA, than without. There was a near-parallel shift in the curves and no significant changes in the slopes of the curves for the tumors treated without a ^10^B-carrier or with BPA or BSH. This indicates that no apparent radio-sensitizing or -protecting effect was observed with or without bevacizumab, nicotinamide or MTH in terms of the numbers of lung metastases. However, with irradiation, nicotinamide reduced the numbers of metastatic nodules from the local tumors treated with the neutron beam only, BPA-BNCT, or BSH-BNCT. The combination with bevacizumab also reduced the number of metastases from the local tumors treated with MTH more than those with nicotinamide and without nicotinamide or MTH.

The numbers of lung metastases from local tumors that received irradiation under each set of conditions, which produced an identical SF of 0.3 as an initial effect (Fig. 1), were estimated using the data shown in Fig. 3 (Table VII). Overall, BNCT with a ^10^B-carrier, especially BPA, decreased the number of metastases more than neutron beam irradiation only. Irrespective of a ^10^B-carrier, irradiation in combination with nicotinamide resulted in a smaller number of metastases than any other combination. Further combination with bevacizumab produced further decreased numbers.

## Discussion

The cellular distribution of ^10^B from BSH is considered to be mostly dependent on the diffusion of the drug, whereas that from BPA is more dependent on the ability of the cells to take up ^10^B (2). Q cell populations have been demonstrated to have a much larger hypoxic fraction (HF) than total cell populations (11). As hypoxic cells are thought to exhibit less uptake than aerobic cells (24), it follows that Q cells have a lower uptake capacity than the total cell population, and that the distribution of ^10^B from ^10^B-carriers into Q cells is more dependent on the diffusion of the drugs than on the uptake ability of the cells.

Perfusion-related acute hypoxia is caused by inadequate blood flow in tissues. Tumor microvasculature frequently has severe structural and functional abnormalities, such as a disorganized vascular network, dilations, an elongated and tortuous shape, an incomplete endothelial lining, a lack of physiological/pharmacological receptors, an absence of flow regulation, and intermittent stasis (25). Perfusion-related O_2_ delivery leads to ischemic hypoxia, which is often transient. Thus, acute hypoxic areas are distributed throughout the tumor depending on these causative factors (8,10,14). Nicotinamide, a vitamin B3 analog, prevents these transient fluctuations in tumor blood flow that lead to the development of acute hypoxia (26). Diffusion-related chronic hypoxia is caused by an increase in diffusion distances with tumor expansion. This results in an inadequate O_2_ supply for cells distant (>70 μm) from the nutritive blood vessels. Diffusion-related hypoxia may also be caused by the deterioration of diffusion ‘geometry’ for example, concurrent versus countercurrent blood flow within the tumor microvessel network (10,24). MTH prior to irradiation decreased the HF, even when combined with nicotinamide administration. By contrast, in a previous study, MTH did not decrease the HF when tumor-bearing mice were placed in a circulating carbogen (95% O_2_/5% CO_2_) chamber during irradiation (11). Thus, MTH has been demonstrated to increase the tumor response to radiation by improving tumor oxygenation through an increase in tumor blood flow (27), thereby preferentially overcoming chronic hypoxia rather than acute hypoxia. Furthermore, the previous finding that the HFs in the total and Q cell populations of B16-BL6 tumors are predominantly composed of acute and chronic HFs, respectively needs to be taken into account (12).

The recombinant humanized monoclonal antibody, bevacizumab is composed of the human immunoglobulin G 1 (IgG1) framework regions and the antigen-binding regions from the murine IgG1 anti-human VEGF monoclonal antibody (28). In previous animal experiments, tumor hypoxia decreased two days following antiangiogenic treatment, such as VEGF-blocking therapy, was almost absent by day five, and increased again by day eight (7,15). In addition to reducing hypoxia, antiangiogenic treatment is considered to be associated with the recruitment of pericytes that help to support vessel walls to the tumor blood vessel. This stabilizes the broken and dilated vasculature that is a common characteristic of tumor vessels (29). Pericyte-covered vessels have also been reported to decrease in number by day eight following antiangiogenic treatment (28). Vascular normalization including the recruitment of pericytes is thought to occur 2–5 days following the blocking of VEGF (1,3). During this window, pericyte coverage of tumor vessels (11,12) and a reduction in tumor vessel permeability and interstitial fluid pressure (30) occur, resulting in the normalization of the tumor vessels leading to a release from acute hypoxia. The decrease in the HFs induced by combining bevacizumab with MTH was more marked than that achieved by combining bevacizumab with nicotinamide treatment in both the total and Q cell populations.

As shown in Table II, concerning the distribution of ^10^B in the total cell population within tumors, a minor improvement was achieved through the chronic hypoxia-releasing treatment MTH when BPA, which delivers more ^10^B to normoxic total tumor cells, rather than BSH, was used. By contrast, a small improvement was achieved through the acute hypoxia-releasing agent nicotinamide or bevacizumab when BSH, which delivers more ^10^B to hypoxic Q cells than BPA, was used. Regardless of which ^10^B-carrier was used, the ^10^B concentration in tumors may be raised by combining the ^10^B-carrier treatment with a treatment that is able to efficiently release the hypoxic areas to which the distribution of ^10^B from each ^10^B-carrier does not readily occur.

The data in table III supports this as when bevacizumab was not used, the distribution of ^10^B in the tumor from BSH relied mostly on passive diffusion, whereas that from BPA relied on uptake capacity in the tumor by active transport. The former resulted in a greater effect on Q cells and the latter, on the total tumor cell population. When bevacizumab was used, the increase in the enhancement ratio was greater in the total cells and with the use of BPA than that in the Q cells and with the use of BSH, respectively. This suggests that bevacizumab was able to efficiently release the acute hypoxia, resulting in a higher uptake of ^10^B from BPA than from BSH into oxygenated tumor cells originating from a state of acute hypoxia. Furthermore, in Table IV, when bevacizumab was used, the increase in the enhancement ratio was more marked in the total cells, with the use of BPA and with MTH, than in the Q cells, with the use of BSH and with nicotinamide, respectively.

Regardless of the presence of bevacizumab, the sensitivity-enhancing effect of nicotinamide and MTH on the total cell population (Table V) almost paralleled the changes in the ^10^B concentration in tumors shown in Table II. Thus, MTH combined with BPA, and nicotinamide combined with BSH induced a greater sensitivity-enhancing effect on the total cell population. In the Q cell population, MTH induced a significantly greater enhancing effect (P<0.05), regardless of which ^10^B-carrier was used. When bevacizumab was combined, the effect of the nicotinamide combination was reduced as both nicotinamide and bevacizumab demonstrated a similar acute hypoxia-releasing effect. However, the combination with bevacizumab had almost no influence on the effect of MTH since the former and the latter exhibited respective acute and chronic hypoxia-releasing effects independently. Based on these findings, the difference in sensitivity between total and Q cell populations increased with nicotinamide or bevacizumab and decreased with MTH (Table VI). Furthermore, the fact that the employed reactor neutron beams included not only high linear-energy-transfer (LET) neutrons but also low LET γ-rays, may have meant that even when a ^10^B-carrier was not used, nicotinamide or bevacizumab and MTH had a marginal sensitivity-enhancing effect on the total and Q cell populations, respectively. Thus, even without a ^10^B-carrier, the difference in sensitivity between the total and Q cell populations increased with nicotinamide or bevacizumab and decreased with MTH (Table VI). Since both nicotinamide and bevacizumab demonstrated a similar acute hypoxia-releasing effect, the difference in sensitivity was not changed significantly in combination with bevacizumab. By contrast, since bevacizumab and MTH induced respective acute and chronic hypoxia-releasing effects independently, the difference in sensitivity became clearer in combination with bevacizumab than without.

In a previous study BNCT was performed, in combination with thalidomide as an antiangiogenic drug, on a short-term basis in order to induce a window of vascular normalization in the treatment of tumors in a hamster cheek pouch model of oral cancer. The blood vessel normalization was not performed to increase the total ^10^B-carrier uptake, but to distribute the ^10^B-carriers effectively to a larger proportion of the tumor cells by fixing the flawed delivery system (31). In particular, pretreatment with thalidomide did not increase the absolute boron content in oral tumors but improved boron targeting homogeneity. Thus, the effect of tumor blood vessel normalization in BNCT remains to be clarified in future studies.

The presence of Q cells is most likely due, at least in part, to hypoxia and the depletion of nutrition as a consequence of poor vascular supply (11,30). As a result, Q cells are viable and clonogenic, but have ceased dividing. This may promote the formation of micronuclei at 0 Gy in Q tumor cells (Table I). Q cells have been revealed to have significantly less radiosensitivity than the total cell population (11,30,32). This may also be applicable to BNCT as more Q cells survive BNCT than do P cells (P<0.05; Fig. 2, Table I). Thus, the control of chronic hypoxic Q cells has a significant impact on the outcome of BNCT for controlling local tumors, resulting in the superiority of BSH as a ^10^B-carrier in BNCT due to the delivery of more ^10^B from BSH in the Q cell population than from BPA. With or without a ^10^B-carrier in the boron neutron capture reaction, nicotinamide and bevacizumab enhanced the radiosensitivity of the total cell population and MTH enhanced the sensitivity of Q cell populations. As a result, the use of nicotinamide or bevacizumab led to an increase, while the use of MTH led to a reduction, in the difference in radiosensitivity (Table VI). Although the use of a ^10^B-carrier in BNCT, especially BPA, significantly increased the difference in radiosensitivity between total and Q cell populations (P<0.05), MTH is considered to be more useful than nicotinamide or bevacizumab in terms of local tumor response since it reduces the difference in radiosensitivity between radiosensitive total and radioresistant Q cell populations. Overall, the use of BSH as a ^10^B-carrier in combination with MTH is considered to be advantageous and promising in terms of local tumor response in BNCT.

Hypoxia is believed to enhance metastasis by increasing genetic instability (10). Acute, but not chronic, hypoxia has been reported to increase the number of macroscopic metastases in mouse lungs (9,10). A previous study reported the significance of injecting an acute hypoxia-releasing agent, nicotinamide, into tumor-bearing mice as a combined treatment with γ-ray irradiation to repress lung metastasis (12). With or without irradiation, nicotinamide and bevacizumab appeared to reduce the number of macroscopic metastases in the current study (Fig. 4, Table VII). Without irradiation or bevacizumab, MTH increased the number of metastases, implying that the release from chronic hypoxia is not as important in repressing metastasis as the release from acute hypoxia. However, hyperthermia is not thought to induce metastasis in the clinical setting (33). As the delivered total dose increased with irradiation, the number of macroscopic lung metastases decreased reflecting the decrease in the number of clonogenically viable tumor cells in the primary tumor (Fig. 4). The metastasis-repressing effect achieved through a reduction in the number of clonogenic tumor cells by irradiation is much greater than that achieved by releasing tumor cells from acute hypoxia. However, more ^10^B from BPA than from BSH could be distributed into the acute hypoxic total tumor cell population, resulting in a greater reduction in the number of highly clonogenic P tumor cells with BPA-BNCT than with BSH-BNCT and with neutron beam irradiation only. BPA-BNCT rather than BSH-BNCT has certain potential to decrease the number of lung metastases and an acute hypoxia-releasing treatment, such as the administration of nicotinamide or bevacizumab, may be promising for reducing the number of lung metastases. Consequently, BPA-BNCT in combination with nicotinamide and/or bevacizumab treatment may demonstrate a higher potential in reducing the number of metastases. Finally, it was revealed that control of the chronic hypoxic Q cell population in the primary solid tumor has the potential to impact the control of local tumors as a whole and that control of the acute hypoxic total tumor cell population in the primary solid tumor has the potential to impact the control of lung metastases.

## Figures and Tables

**Table I:** Plating efficiency and micronucleus frequency at 0 Gy.

Variable	Without nicotinamide or MTH	With nicotinamide	With MTH
Plating efficiency (%)			
Without bevacizumab			
Without ^10^B-carrier	84.4±8.2	81.4±7.3	83.5±8.7
With BPA^c^	76.9±7.7	69.9±6.5	73.9±7.3
With BSH^d^	81.4±8.3	74.9±6.3	78.9±6.8
With bevacizumab			
Without ^10^B-carrier	80.1±8.0	76.3±7.1	78.4±8.3
With BPA	69.6±7.1	64.8±6.2	68.8±7.0
With BSH	74.1±7.7	69.8±7.2	73.8±7.1
Micronucleus frequency			
Without bevacizumab			
Total cell population			
Without ^10^B-carrier	0.050±0.008	0.057±0.006	0.054±0.005
With BPA	0.063±0.008	0.081±0.008	0.077±0.007
With BSH	0.059±0.008	0.078±0.009	0.074±0.008
Quiescent cell population			
Without ^10^B-carrier	0.077±0.008	0.084±0.009	0.081±0.009
With BPA	0.091±0.009	0.110±0.011	0.105±0.010
With BSH	0.095±0.009	0.120±0.011	0.115±0.011
With bevacizumab			
Total cell population			
Without ^10^B-carrier	0.058±0.008	0.068±0.007	0.069±0.007
With BPA	0.071±0.008	0.092±0.009	0.092±0.009
With BSH	0.067±0.008	0.089±0.009	0.089±0.009
Quiescent cell population			
Without ^10^B-carrier	0.083±0.008	0.086±0.009	0.087±0.009
With BPA	0.097±0.009	0.112±0.011	0.111±0.011
With BSH	0.101±0.009	0.122±0.011	0.121±0.011

Data are presented as mean ± standard error (n=9). MTH, mild temperature hyperthermia; BPA, l-*para*-boronophenylalanine-^10^B; BSH, sodium mercaptoundecahydrododecaborate-^10^B.

**Table II:** ^10^B concentration (μg/g=ppm) in tumors and boron dose rate (cGy/h).

Variable	Without nicotinamide or MTH	With nicotinamide	With MTH
^10^B concentration (μg/g)			
Without bevacizumab			
BPA	6.9±0.8	7.3±1.0	8.3±1.1
BSH	7.2±0.9	8.4±1.1	7.9±1.0
With bevacizumab			
BPA	7.2±0.9	7.4±1.1	8.6±1.2
BSH	8.0±1.0	8.6±1.2	8.7±1.2
Boron dose rate (cGy/h)			
Without bevacizumab			
BPA	184.2±21.4	194.9±26.7	221.6±29.4
BSH	192.2±24.0	224.3±29.4	210.9±26.7
With bevacizumab			
BPA	192.2±24.0	197.6±29.4	229.6±32.0
BSH	212.7±26.7	229.6±32.0	231.3±32.0

Data are presented as mean ± standard error (n=9). MTH, mild temperature hyperthermia; BPA, L-*para*-boronophenylalanine-^10^B; BSH, sodium mercaptoundecahydrododecaborate-^10^B.

**Table III:** Enhancement ratios^a^ due to combination with a ^10^B-carrier.

	^10^B-carrier	
	
Variable	BPA	BSH
Surviving fraction = 0.3		
Total cell population		
Without bevacizumab	1.7±0.1b,c	1.5±0.1^c^
With bevacizumab	2.0±0.15b,d	1.65±0.15^d^
Net micronucleus frequency = 0.3		
Total cell population		
Without bevacizumab	2.45±0.15	2.25±0.15
With bevacizumab	2.6±0.2	2.35±0.15
Quiescent cells		
Without bevacizumab	1.5±0.1^e^	1.8±0.1^e^
With bevacizumab	1.6±0.15	1.85±0.15

Data are presented as mean ± standard error (n=9).

a The ratio of the dose of radiation necessary to obtain each end-point without a ^10^B-carrier to that needed to obtain each end-point with a ^10^B-carrier; BPA, L-*para*-boronophenylalanine-^10^B; BSH, sodium mercaptoundecahydrododecaborate-^10^B;

b,c,d,e Differences between two values labeled with the same letter are significant (P<0.05).

**Table IV:** Enhancement ratios^a^ due to combined treatment with bevacizumab.

Variable	Without nicotinamide or MTH	With nicotinamide	With MTH
Surviving fraction = 0.3			
Total cell population			
Without ^10^B-carrier	1.25±0.1	1.05±0.1	1.3±0.1
With BPA	1.55±0.2	1.05±0.1	1.7±0.15
With BSH	1.35±0.1	1.05±0.15	1.35±0.15
Net micronucleus frequency = 0.3			
Total cell population			
Without ^10^B-carrier	1.1±0.1	1.05±0.1	1.2±0.1
With BPA	1.2±0.2	1.05±0.1	1.3±0.15
With BSH	1.15±0.1	1.05±0.1	1.25±0.15
Quiesent cell population			
Without ^10^B-carrier	1.05±0.1	1.05±0.1	1.05±0.1
With BPA	1.05±0.2	1.05±0.1	1.1±0.1
With BSH	1.1±0.1	1.05±0.1	1.2±0.1

Data are presented as mean ± standard error (n=9).

a Ratio of the dose of radiation necessary to obtain each end-point without bevacizumab to that needed to obtain each end-point with bevacizumab;

MTH, mild temperature hyperthermia; BPA, L-*para*-boronophenylalanine-^10^B; BSH, sodium mercaptoundecahydrododecaborate-^10^B.

**Table V:** Enhancement ratios^a^ due to combined treatment with nicotinamide or mild temperature hyperthermia.

Variable	Nicotinamide	MTH
Surviving fraction = 0.3		
Total cell population		
Without bevacizumab		
Without ^10^B-carrier	1.3±0.15	1.2±0.1
With BPAc	1.25±0.1	1.4±0.15
With BSHd	1.5±0.15	1.2±0.1
With bevacizumab		
Without ^10^B-carrier	1.2±0.1	1.1±0.1
With BPA	1.15±0.1	1.35±0.15
With BSH	1.3±0.15	1.15±0.1
Net micronucleus frequency = 0.3		
Total cell population		
Without bevacizumab		
Without ^10^B-carrier	1.25±0.1	1.15±0.1
With BPA	1.15±0.1	1.3±0.1
With BSH	1.4±0.1	1.2±0.1^b^
With bevacizumab		
Without ^10^B-carrier	1.1±0.1	1.15±0.1
With BPA	1.1±0.1	1.2±0.1
With BSH	1.2±0.1	1.15±0.1^c^
Quiescent cell population		
Without bevacizumab		
Without ^10^B-carrier	1.1±0.1	1.2±0.1
With BPA	1.15±0.1^d^	1.4±0.1^d^
With BSH	1.2±0.1^e^	1.45±0.15b,e
With bevacizumab		
Without ^10^B-carrier	1.05±0.1	1.15±0.1
With BPA	1.1±0.1^f^	1.35±0.15^f^
With BSH	1.15±0.1^g^	1.4±0.15c,g

Data are presented as mean ± standard error (n=9).

a Ratio of the dose of radiation necessary to obtain each end-point without combined treatment to that needed to obtain each end-point with the combined treatment; MTH, mild temperature hyperthermia; BPA, L-*para*-boronophenylalanine-^10^B; BSH, sodium mercaptoundecahydrododecaborate-^10^B;

b–g Differences between two values labeled with the same letter are significant (P<0.05).

**Table VI:** Dose-modifying factors for quiescent cells relative to the total cell population^a^ (net micronucleus frequency = 0.3).

Variable	Without nicotinamide or MTH	With nicotinamide	With MTH
Without bevacizumab			
Without ^10^B-carrier	1.3±0.1	1.45±0.1	1.25±0.1
With BPA	2.1±0.2	2.35±0.2	1.7±0.15
With BSH	1.6±0.15	1.75±0.15	1.25±0.1
With bevacizumab			
Without ^10^B-carrier	1.4±0.1	1.5±0.15	1.4±0.1
With BPA	2.3±0.2	2.45±0.2	2.1±0.2
With BSH	1.65±0.15	1.75±0.15	1.45±0.1

Data are presented as mean ± standard error (n=9).

a Ratio of the dose of radiation necessary to obtain each end-point in the quiescent cell population to that needed to obtain each end-point in the total tumor cell population;

MTH, mild temperature hyperthermia; BPA, L-*para*-boronophenylalanine-^10^B; BSH, sodium mercaptoundecahydrododecaborate-^10^B.

**Table VII:** Numbers of metastases from the irradiated tumors that received cytotoxic treatment producing a similar initial local effect^a^ (surviving fraction = 0.3).

Variable	Without nicotinamide or MTH	With nicotinamide	With MTH
Without bevacizumab			
Without ^10^B-carrier	17.9	16.6	17.9
With BPA	15.3	14.5	15.1
With BSH	15.9	15.2	15.5
With bevacizumab			
Without ^10^B-carrier	17.4	16.3	16.8
With BPA	14.5	14.2	14.3
With BSH	15.6	14.8	15.4

a Based on the data shown in Fig. 4, the estimated numbers of lung metastatic nodules from local tumors that received neutron beam irradiation with or without a ^10^B-carrier in combination with nicotinamide or mild temperature hyperthermia, which produced an identical surviving fraction of 0.3 as an initial effect on Fig. 1;

MTH, mild temperature hyperthermia; BPA, L-*para*-boronophenylalanine-^10^B; BSH, sodium mercaptoundecahydrododecaborate-^10^B.
